# Temporal bone manifestation of primary extranodal Rosai–Dorfman disease: a case report

**DOI:** 10.1186/s13256-023-03790-8

**Published:** 2023-06-21

**Authors:** E. Koonar, F. Ramazani, M. Hyrcza, J. Chau

**Affiliations:** 1 Division of Otolaryngology-Head and Neck Surgery, Department of Surgery, Cumming School of Medicine, University of Calgary, Calgary, Canada; 2 Department of Pathology and Laboratory Medicine, Arnie Charbonneau Cancer Institute, University of Calgary, Calgary, Canada

**Keywords:** Temporal bone, Rosai–Dorfman disease, Histiocytosis, Case report

## Abstract

**Background:**

Rosai–Dorfman disease is a rare benign histiocytic disorder characterized in most cases by painless cervical adenopathy. Less than 10% of extranodal cases involve bony lesions. Primary bone Rosai–Dorfman disease in the absence of nodal disease is extremely rare.

**Case presentation:**

A 48 year-old Caucasian male presented with progressive right-sided otalgia, tinnitus, vertigo, and hearing loss. A right temporal bone lytic lesion was detected on diagnostic imaging. Resection of the lesion and histopathological examination revealed Rosai–Dorfman disease.

**Conclusions:**

Rosai–Dorfman disease primary bone lesions are an atypical presentation of a rare disease. This is the second reported case of Rosai–Dorfman disease arising within the temporal bone. This case study reveals that Rosai–Dorfman disease should be considered for patients presenting with inflammatory/lytic lesions of the temporal bone, in cases where infection and malignancy have been excluded.

## Background

Rosai–Dorfman disease (RDD) is a rare, benign, non-Langerhans cell histiocytosis. While most patients present with massive bilateral painless cervical lymphadenopathy [1], cases of mediastinal, axillary, and inguinal lymphadenopathy have also been described. Secondary extranodal localization of disease involving the skin, soft tissue, gastrointestinal tract, or central nervous system [2] has been reported in 43% of cases. Primary bone RDD in the absence of lymphadenopathy accounts for less than 1% of cases [3]. Bone involvement presents radiographically as lytic lesions with well-defined sclerotic margins, and occurs in less than 10% of cases [4]. Diagnosis of RDD depends on histopathologic features, including the finding of emperipolesis, along with immunoprofile of histiocytes showing positivity for S100 and CD68, and lacking CD1a expression. There are several postulated etiologies of RDD, none of which has been definitively proven [5]. Recent research has shown frequent mutations in the mitogen-activated protein kinase (MAPK) pathway genes [6, 7], as well as consistent cyclin D1 activation [8].

## Case presentation

A 48 year-old Caucasian male presented with an 18-month history of progressive right-sided otalgia, nonpulsatile tinnitus, bouts of vertigo, and unilateral hearing loss. He had no previous history of recurrent otitis media, ototoxic medication exposure, loud noise exposure, or chemotherapy. On physical examination, he was found to have narrowing and inflammation of the external auditory canal (EAC), as well as tenderness of the mastoid bone to palpation without fluctuation. The tympanic membrane was not visualized due to the chronic inflammation and narrowing of the EAC. There was no evidence of cervical lymphadenopathy.

**Fig. 1:**
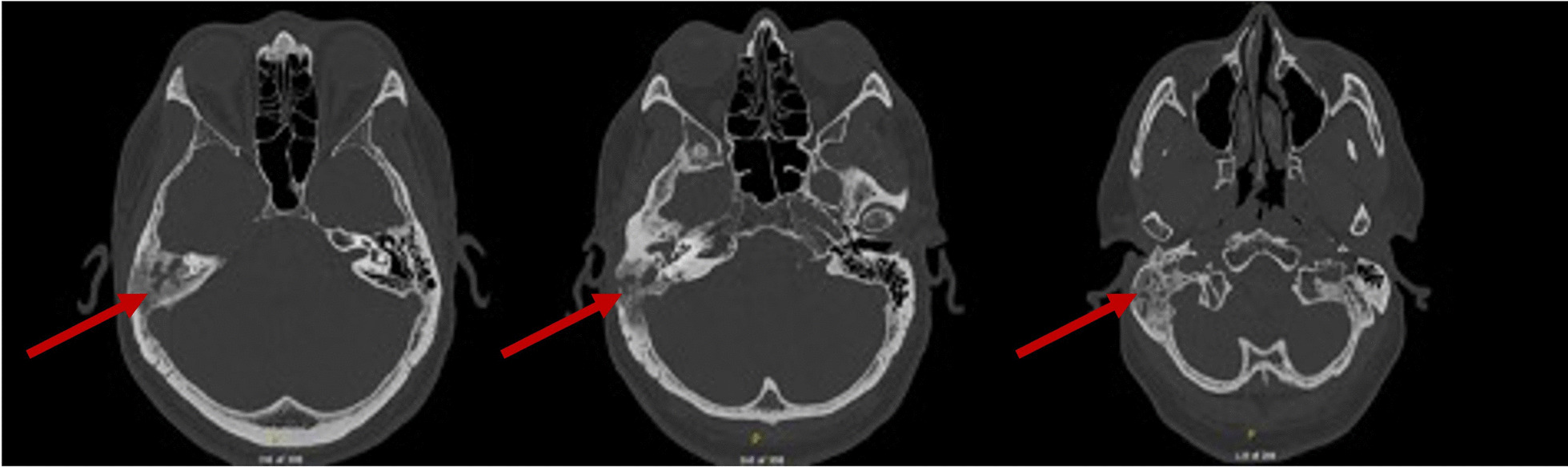
Three axial images of computed tomography temporal bone with arrow showing a lytic lesion in the right mastoid cavity

A dedicated computed tomography (CT) scan of the temporal bones demonstrated diffuse opacification throughout the right temporal bone, with airspace coalescence and lytic destructive changes involving the mastoid portion of the temporal bone. The destruction involved the outer cortex of the mastoid bone with surrounding thickening and sclerosis of the mastoid air cells. The opacification extended throughout the middle ear cavity into the epitympanum, Prussak’s space, facial recess, and sinus tympani. There was soft tissue thickening surrounding the EAC, causing near-complete stenosis (Fig. 1).

**Fig. 2:**
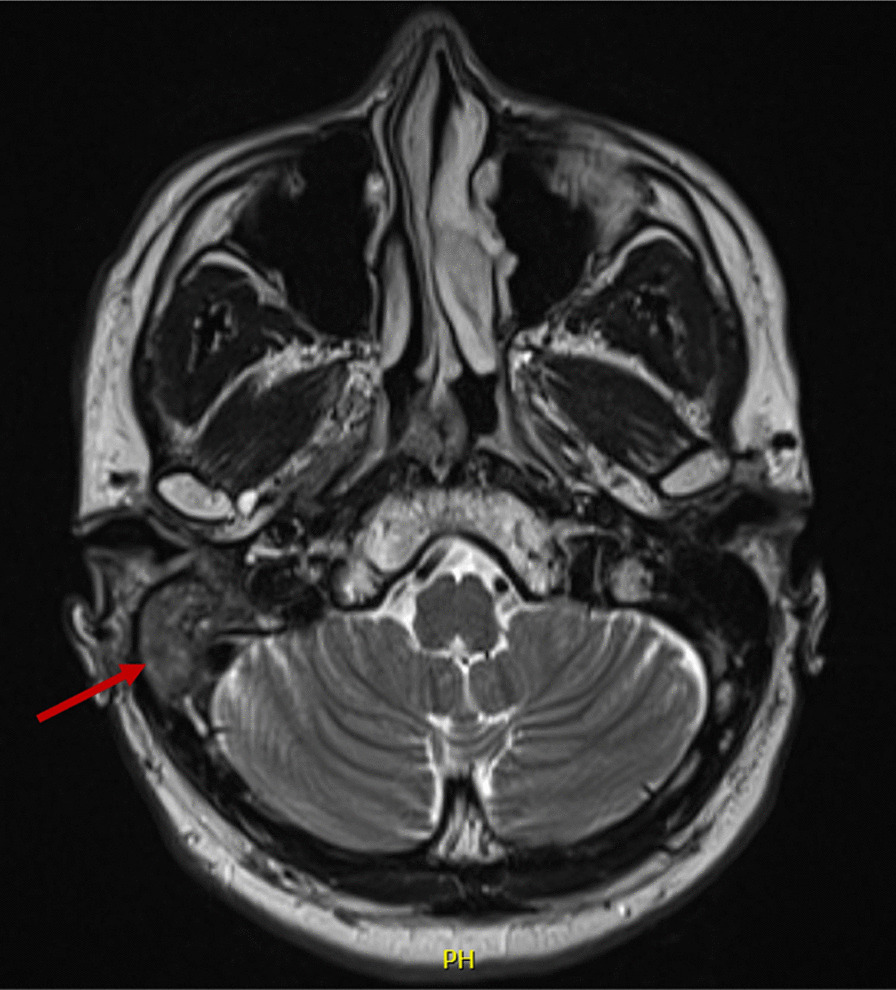
T2-weighted enhanced brain magnetic resonance imaging with arrow showing a right-sided soft tissue lesion within the mastoid cavity

Enhanced brain magnetic resonance imaging (MRI) showed a right-sided mastoid lesion with dural thickening superior and posterior to the petrous temporal bone, as well as some minor enhancing disease in the inferior skull base around the right styloid process. There was also abnormal enhancement seen in the vestibular aqueduct, which extended inferiorly to involve the dura, and about the medial margin of the sigmoid sinus. A nonenhancing/hypoenhancing plug of soft tissue was noted within the medial EAC that abutted the tympanic membrane, along with abnormal enhancement of the middle ear cavity (Fig. 2).

**Fig. 3:**
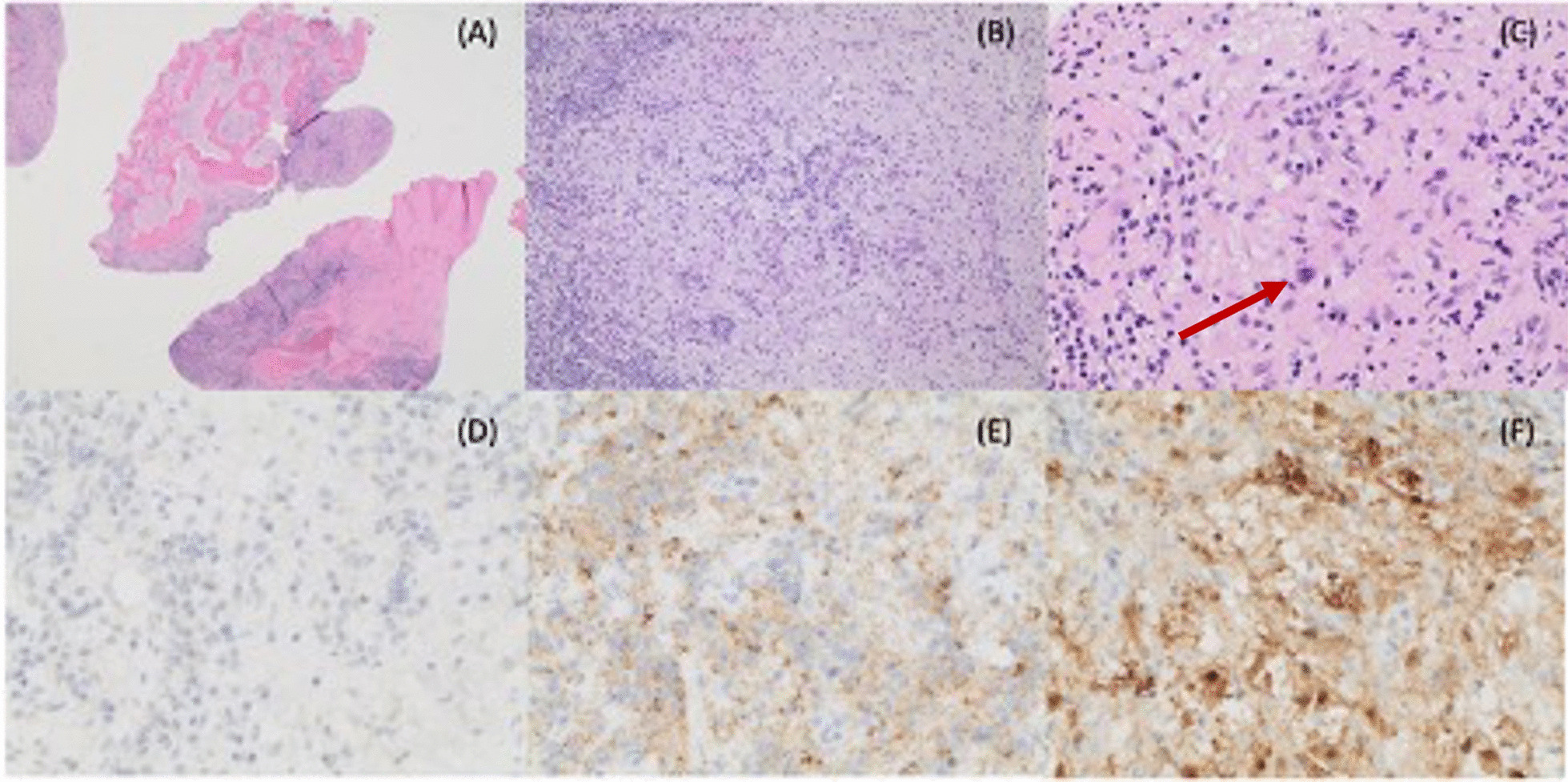
Hematoxylin and eosin (H&E) staining of biopsy taken from right mastoid bone at 20× (**A**), 100× (**B**), and 400× (**C**). The higher magnification shows a prominent population of histiocytes, as well as evidence of emperipolesis (arrow showing a histiocyte with emperipolesis) with engulfment of lymphocytes. Immunohistochemistry staining of biopsy taken from right mastoid bone shows that the atypical histiocytes within the specimen are negative for CD1a (**D**, 400×) and positive for CD68 (**E**, 400×), with coexpression of S100 (**F**, 400×)

The initial trans-external canal biopsy was inconclusive, showing only granulation tissue keratinizing squamous epithelium. Subsequent open mastoid biopsy was performed during which squamous debris was found filling the medial ear canal. There was also a significant area of mastoid cortical breach, with granulation-like tissue extending through the mastoid cortex and filling the visualized mastoid cavity. The squamous debris and granulation tissue within the mastoid ear cavity was debrided. Pathology showed lymphohistiocytic infiltrate surrounding residual bony trabeculae. The lymphoid cells were polymorphic and consisted primarily of T cells, with admixed B cells and plasma cells. The histiocytes contained abundant eosinophilic granular cytoplasm and ovoid nuclei. Nucleoli were inconspicuous, mitotic rate was low, and no necrosis was identified. The sample histiocytic component showed positivity for CD68 and S100 by immunohistochemistry, and lack of CD1a expression (Fig. 3). A tentative diagnosis of Rosai–Dorman disease was made.

The patient remained symptomatic, with headaches, nonspecific visual changes, and histiocytosis. A trial of oral corticosteroids (prednisone 1 mg/kg for a week followed by a taper) was prescribed after consultation with the rheumatology and hematologic oncology services, with no overall impact on the patient’s symptoms. The patient agreed to another surgical intervention. A cortical mastoidectomy was performed under general anesthetic. The mastoid air cells, mastoid antrum, and epitympanum were filled with granulation-like tissue without evidence of abscess or cholesteatoma. Pathology confirmed Rosai–Dorfman disease.

The patient recovered without complications and noted a significant improvement in his headache, visual field changes, and histiocytosis at his 1-week postoperative visit. At his most recent follow-up 6 months post-mastoidectomy, the patient had returned to work and was functioning at a near-normal level. Rheumatologic investigations to assess other sites of extranodal disease were performed, but none was found.

## Discussion

Rosai–Dorfman disease (RDD) is characterized by idiopathic histiocytic proliferation, most commonly presenting in the cervical lymph nodes. RDD is a rare disease with a prevalence of 1:200,000 and an estimated 100 new cases per year in the USA [9]. It is more frequently seen in young adults (mean age, 20.6 years), although it has been reported in patients 5–74 years of age. RDD is more common in males and in individuals of African descent [9].

The etiology of Rosai–Dorfman disease is unclear; however, several hypotheses have been proposed as causes of this disease: viral infection by human herpesvirus 6 and Epstein–Barr virus [10], immunological disorders, and neoplastic transformation of histiocytes. This last hypothesis has recently been bolstered by the discoveries of recurrent mutations in the MAPK pathway genes [6, 7], as well as consistent Cyclin D1 activation [8].

Early descriptions of the disease focused on the triad of (1) massive cervical lymphadenopathy, (2) expanded lymph node sinuses, and (3) histiocytosis with emperipolesis, that is, the presence of variable numbers of intact lymphocytes within the cytoplasm of the lesional histiocytes [1, 5]. Subsequent reports have revealed that extranodal disease occurs in approximately 43% of cases [2]. The skin is involved in 10% of RDD cases, though isolated cutaneous RDD is rare. Lesions have been described as slow-growing, painless, nonpruritic nodules, plaques, or papules, with coloration varying from yellow to red to brown [10]. Central nervous system (CNS) involvement of RDD has been reported in < 5% of cases, with 75% occurring as intracranial and 25% as spinal lesions. Patients with CNS involvement often do not present with lymphadenopathy [11]. Ophthalmic disease occurs in 11% of RDD cases [3] and presents as a mass in the orbital soft tissues. There are additional rare reported manifestations of the disease, including intrathoracic (2% of patients) [12], kidney (4% of patients) [13], and gastrointestinal (< 1% of cases) [3].

Within the head and neck, involvement of the nasal cavity and paranasal sinuses is the most common, occurring in 11% of RDD cases [3]. This generally presents as epistaxis, nasal dorsum deformity, facial asymmetry, or aural fullness. Oral cavity involvement, though rare, can also occur and includes the mucosa of the oropharynx, tonsils, salivary and parotid glands, larynx, pharynx, and thyroid gland, which present as recurrent enlargement and can cause symptoms due to mass effect [14, 15].

Mosheimer *et al.* reviewed 108 patients with RDD with bone involvement and reported that primary RDD of the bone was observed in 67 (74.4%) cases. The most common sites of primary bone RDD are the cranium (30.6%), the facial bones (22.2%), and the tibia (17.6%) [14]. Primary bone RDD usually occurs in the absence of lymphadenopathy, while the compression of the histiocytes in the intertrabecular spaces makes the demonstration of the emperipolesis difficult and in fact often makes the entire histiocytic component unapparent and, therefore, easy to overlook. The lack of classical histology of RDD makes this diagnosis easy to miss [11]. The differential diagnosis of RDD in the bone is extensive and includes osteomyelitis, Langerhans cell histiocytosis and other histiocytosis, lymphoma, immunoglobulin 4 (IgG4)-related disease, primary bone sarcoma, and lytic bone metastases [12]. Therefore, accurate diagnosis depends on histological examination. Immunohistochemistry is key in confirming the diagnosis, with the large pale histiocytes of Rosai–Dorfman disease staining for S100, CD68, and vimentin, but showing lack of CD1a, keratin, and CD45 expression [5].

In the current literature, there is one previously reported case of RDD arising in the temporal bone. The case, described by Allegranza *et al.*, was that of a 14-year-old girl who presented with firm, painless swelling in the parietotemporal region. Surgical resection revealed a tumor measuring 2 cm in diameter, which had eroded into the right temporal bone, and was slightly adherent to, but not infiltrating, the dura mater. The patient underwent curettage of the bone and was reported to be disease free 17 months after treatment [15].

Demicco *et al.* reported the clinical course of primary RDD of bone, demonstrating that 42% of patients developed additional extraosseous manifestations, including testicular, lymph node, and subcutaneous lesions. One patient developed multiple additional bone lesions without extraosseous disease. These additional lesions developed from 3 months to 3 years after initial treatment. Therefore, at least 3 years of follow-up may be necessary to detect the development of additional lesions [15].

On imaging, Raslan *et al.* reported that the affected areas are usually homogeneously enhancing and might show central hypodensity on CT. MRI characteristics of the involved areas are generally T1 isointensity, T2 hyperintensity, and intense enhancement with gadolinium agents [13].

The treatment for RDD is varied, and optimal treatment has yet to be established. Among all patients with RDD, 20% show spontaneous remission without therapy [1]. Reported treatments for RDD include intralesional and oral administration of corticosteroids, chemotherapy, low-dose interferon, antibiotic therapy, radiation therapy, and surgery. Intensive treatment is generally reserved for disease manifestations within vital organs, including the central nervous system, vasculature, orbit, and nasal cavity [1]. In the present case, the patient underwent surgical excision of the tumor and debridement of the mastoid bone.

## Conclusions

This is the second reported case of confirmed Rosai–Dorfman disease arising in the temporal bone. This study suggests that RDD can mimic infiltrative/inflammatory lesions of the temporal bone and should be considered in the differential diagnosis for patients with unilateral hearing loss and inflammatory/lytic lesions on diagnostic imaging, when infectious and malignant disorders have been ruled out.

## Data Availability

The authors confirm that the data supporting the findings of this study are available within the article and/or its additional materials.

## References

[1] Rosai J, Dorfman R (1969). Sinus histiocytosis with massive lymphadenopathy. Arch Pathol.

[2] Gaitnode S (2007). Multifocal, extranodal sinus histiocytosis with massive lymphadenopathy: an overview. Arch Pathol Lab Med.

[3] Foucar E, Rosai J, Dorfman R (1990). Sinus histiocytosis with massive lymphadenopathy (Rosai–Dorfman disease): review of the entity. Semin Diagn Pathol.

[4] Bruce-Brand C, Schneider JW, Schubert Pl (2020). Rosai–Dorfman disease: an overview. J Clin Pathol.

[5] Dalia S, Sagatys E, Sokol L, Kubal T (2014). Rosai–Dorfman disease: tumor biology, clinical features, pathology, and treatment. Cancer Control.

[6] Garces S, Medeiros L, Patel K, Li S, Pina-Oviedo S, Li J, Garces J, Khoury J, Yin C (2017). Mutually exclusive recurrent KRAS and MAP2K1 mutations in Rosai–Dorfman disease. Mod Pathol.

[7] Barabaran E, Sadigh S, Rosenbaum J, Arnam JV, Bogusz A, Mehr C, Bagg A (2019). Cyclin D1 expression and novel mutational findings in Rosai–Dorfman disease. Br J Haematol.

[8] Garces S, Medeiros L, Marques-Piubelli M, Coelho Siqueira S, Miranda R, Cuglievan B, Sriganesha V, Medina A, Garces J, Saluja K, Bhattacharjee M, Khoury J, Li S, Xu J, Jelloul F, Thakral B, Cameron YC (2022). Cyclin D1 expression in Rosai–Dorfman disease: a near constant finding that is not invariably associated with MAPK/ERK pathway activation. Hum Pathol.

[9] Garcia R, DiCarlo E (2022). Rosai–Dorfman disease of bone and soft tissue. Arch Pathol Lab Med.

[10] Baker J, Kyriakos M, McDonald DJ, Rubin DA (2017). Primary Rosai–Dorfman disease of the femur. Skeletal Radiol..

[11] Raslan O, Schellingerhout D, Fuller G, Ketonen L (2011). Rosai–Dorfman disease in neuroradiology: imaging and findings in a series of 10 patients. Am J Roentgenol.

[12] Mosheimer B, Oppl B, Zandieh S, Fillitz M, Keil F, Klaushofer K, Weizz G, Zwerina J (2017). Bone involvement in Rosai–Dorfman disease (RDD): a case report and systematic literature review. Curr Rheumatol Rep.

[13] Allegranza A, Barbareschi M, Solero C, Fornari M, Lasio G (1991). Primary lymphohistiocytic tumour of the bone: a primary osseous localization of Rosai–Dorfman disease. Histopathology.

[14] Lai K, Abdullah V, Ng K, Fung N, Hasselt CV (2013). Rosai–Dorfman disease: presentation, diagnosis, and treatment. Head Neck.

[15] Demicco E, Rosenberg A, Bjornsson J, Rybak L, Unni K, Nielsen G (2010). Primary Rosai–Dorfman disease of the bone: a clinicopathologic study of 15 cases. Am J Surg Pathol.

